# Y-Split Recession of the Medial Rectus Muscle as a Secondary and/or Unilateral Procedure in the Treatment of Esotropia with Distance/Near Disparity

**DOI:** 10.1155/2017/6472690

**Published:** 2017-07-19

**Authors:** Monika Wipf, Siegfried Priglinger, Anja Palmowski-Wolfe

**Affiliations:** ^1^University Eye Hospital, University Basel, Mittlere Strasse 91, 4031 Basel, Switzerland; ^2^Krankenhaus der Barmherzigen Brüder, Linz, Austria; ^3^Allgemeines Krankenhaus der Stadt Linz, Linz, Austria

## Abstract

**Introduction:**

In esotropia with larger angles > near than at distance, splitting of the medial rectus muscle has been suggested as a treatment option. Previous reports of bilateral medial rectus Y-splitting as a first intervention showed a reduction of the distance/near disparity with fewer side effects compared to posterior fixation surgery. We address whether a medial rectus Y-splitting as a secondary and/or a unilateral procedure also reduce distance/near disparity.

**Materials and Methods:**

We retrospectively reviewed the charts of four patients undergoing Y-split recession as a second and/or unilateral surgery. Main outcomes were distance/near disparity and squint angles.

**Results and Discussion:**

Three of the four patients had undergone unilateral Y-splitting of the medial rectus as a secondary surgery, three as a unilateral procedure. Mean distance/near disparity was reduced from 17 PD preoperatively to zero at the final follow-up (FU). Preoperative angles ranged from 45 PD to 66 PD at near and from 25 PD to 55 PD at distance. At the final FU, these angles ranged from 0 PD to 20 PD at near and at distance. Mean FU was 42 months (range: 12–60 months).

**Conclusion:**

Y-split recession as a secondary and/or unilateral surgery for distance/near esotropia can reduce distance/near disparity with good long-term results. Residual esotropia can be corrected by adding resection of the lateral rectus muscle.

## 1. Introduction

In 1991, Bagolini et al. suggested [1] splitting of the medial rectus as an alternative treatment option to the posterior “Faden fixation surgery” suggested by Cüppers [2] in patients with varying angles of esotropia. Since then, other authors have supported the applicability of the Y-splitting procedure in the treatment of esotropia with a larger angle at near than at distance [3–6]. Both surgical procedures can be combined with a recession of the medial rectus muscle. However, in the Y-splitting procedure, the insertion of the medial rectus muscle remains anterior of the equator [3–5], reducing the risk of perforation of the sclera during the operation and retinal detachment. Y-splitting surgery has also been reported to result in less incomitance than posterior fixation surgery [4, 7].

To our knowledge, there has been no previous report on Y-splitting of the medial rectus muscle as a reoperation and/or as a unilateral surgery.

## 2. Materials and Methods

An approval of the Institutional Review Board to undertake this study was obtained in December 2012. The study was conducted in adherence to the Declaration of Helsinki.

We retrospectively reviewed the medical charts of patients who had undergone strabismus surgery between 2008 and 2012. Patients who received Y-split recession of the medial rectus muscle as a secondary or as a unilateral procedure at our institution were identified.

The main outcome measures were the reduction of the distance/near disparity, the squint angle at near and at distance, and adduction and abduction measured with the Kestenbaum Limbus test, whichever is available. We included data collected preoperatively and at postoperative weeks one, twelve, and at the final follow-up (FU).

**Figure 1:**
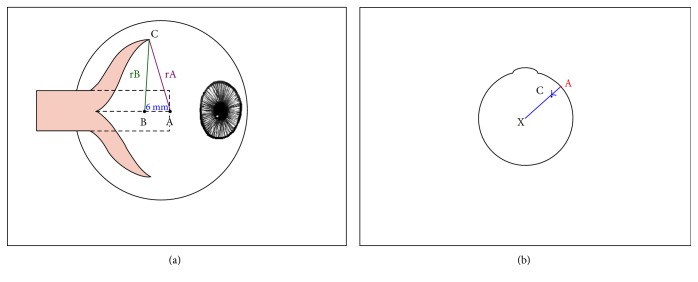
Schematic representation of the Y-splitting procedure of the medial rectus muscle. (a) Side view. (A) point at the middle of the original insertion (dotted line) of the medial rectus muscle. (B) point 6 mm distal of point A. (rA, rB) radius of predetermined distances (see text). Calipers are centered on point A, and a circle is drawn on the sclera with rA; then, the calipers are centered on point B, and a circle with rB is marked on the sclera. The intersection of the circles marks point C, the point of scleral refixation. In this graph, measurements are shown for reattachment of the superior muscle section; the same is applied to the lower half. (b) View of the eye from above. Reattaching the split medial rectus to point C (shown for the superior half) reduces the lever arm (the distance between the center of the globe and the insertion of the muscle), and thus the torque.

The surgical technique is described elsewhere by Hoerantner et al. [4] and is schematically represented in Figure 1. Briefly, the medial rectus muscle is split over 15 mm along its middle and both sides of the muscle are tied with an absorbable suture. The muscle is then detached from its insertion. From the middle of the original insertion (point A) and from point B, which is 6 mm distal to point A, predetermined distances are marked with a radius (rA, rB) to either side of the points. The intersection of the radius paths determines the points of scleral refixation (point C). For bilateral Y-splitting procedures of the medial rectus muscle in an eye with an axial length of 21.2 mm, Priglinger and Hametner suggested rB to be 8 mm, while rA determines the amount of recession added to the procedure. rA varies according to the distance angle, and 3 different dosages are given for angles under 18 prism diopters (PD) (rA = 8.5 mm), for an esotropia between 18 and 27 PD (rA = 9.0 mm), and for angles over 27 PD (rA = 10 mm) (Siegfried Priglinger, personal communication). More detailed dosages may be calculated with a computer program as suggested by Hoerantner et al. [4, 8].

## 3. Results and Discussion

4 patients were identified as having undergone a Y-split recession of the medial rectus muscle as a secondary procedure (*n* = 3) or a unilateral procedure (*n* = 3). At the time of surgery, all patients had worn their full cycloplegic correction for at least 3 months.

Table 1 summarizes patient characteristics, axial lengths, and dosages of surgery.

Overall, the distance/near disparity was 17 PD at baseline and 0 PD at the final FU. Preoperative angles ranged from 45 to 66 PD at near and 25 to 55 at distance. These angles decreased to 0–20 PD at near as well as at distance at the last FU (Table 2).

In the following section, the patients are described in detail.

### 3.1. Patient 1

Patient 1 presented at the age of 23 with typical signs of infantile esotropia: esotropia, latent nystagmus, and dissociated vertical deviation (DVD). The patient could not remember her early strabismus history. At the age of 6 years, she had undergone strabismus surgery on the left eye (no details known), and four years later, the medial rectus muscle of the right eye had been treated with a posterior fixation surgery according to Cüppers. The squint angle, measured with the alternate prism cover test, was 60 PD at near and 45–50 PD at distance. She also had a hypotropia of the right eye of 20 PD at near and at distance. Bagolini was negative at near and at distance. In the following visits, varying angles of 55–66 PD were measured at near, and of 45–55 PD at distance. At the last preoperative visit, the angle at near was 66 PD and 50–55 PD at distance (Table 2). Snellen visual acuity at distance before surgery was 0.8 for the right eye and 1.0 for the left eye.

The forced duction test, performed intraoperatively, revealed a restriction in the abduction of the right eye, which was less severe in the left eye. A unilateral surgery was performed: The right medial rectus muscle was revised and recessed with the Y-splitting procedure, and a 5 mm resection of the right lateral rectus muscle was performed. 1 week later, the angle had decreased to a manifest 30 PD at near and 1 PD at distance. At the last FU at 36 months, the distance/near disparity was resolved. On right fixation, she had an esotropia of 16 PD at near and at distance with a left hypertropia of 25 PD due to the DVD. On left fixation, the angle at near and at distance was 10 PD with a left hypertropia of only 4 PD. Left fixation was assured by fitting the glasses with an additional +2 dpt spherical correction on the right side. At the last FU, the Bagolini test was positive at distance and at near with the right eye performing a corrective outward saccade. The patient was satisfied with the outcome and thus no further treatment was planned.

### 3.2. Patient 2

Patient 2 underwent a Y-split recession at the age of 15 years.

Her congenital esotropia had been treated elsewhere at the age of 2 years with a bilateral medial rectus recession of 7 mm, combined with a bilateral inferior oblique recession. When she first presented to our hospital at the age of 3, years an alternating esotropia and a DVD remained. Under full cycloplegic correction, varying angles up to 12 degrees were measured. As the cosmetic situation was considered satisfactory at conversation distance of 2 meters, emphasis was placed on visual development. She was followed closely and a slight amblyopia of the left eye was successfully treated with occlusion therapy.

At the age of 15 years, the patient opted for further strabismus surgery. Visual acuity at distance and at near was 1.25 OU. Testing of binocularity with the Bagolini test showed suppression of the left eye. At that time, she was under full cycloplegic correction with a squint angle of 50-51 PD at near and 27–29 PD at distance. The angle at near could be reduced to the distance angle by a near addition of +3 dpt. This was, however, not tolerated by the patient. A unilateral Y-split recession of the medial rectus muscle of the left eye was combined with a 5 mm resection of the left lateral rectus muscle. At the last FU at 60 months, distance/near disparity was resolved and only an esophoria of 6 PD remained at distance and at near fixation as well as a small DVD that was well compensated. Surprisingly, binocular functions developed: the Bagolini test was positive at distance and at near with the right eye performing a small corrective saccade from superior. Motility was unimpaired. The patient was satisfied with this long-term result.

### 3.3. Patient 3

**Figure 2:**
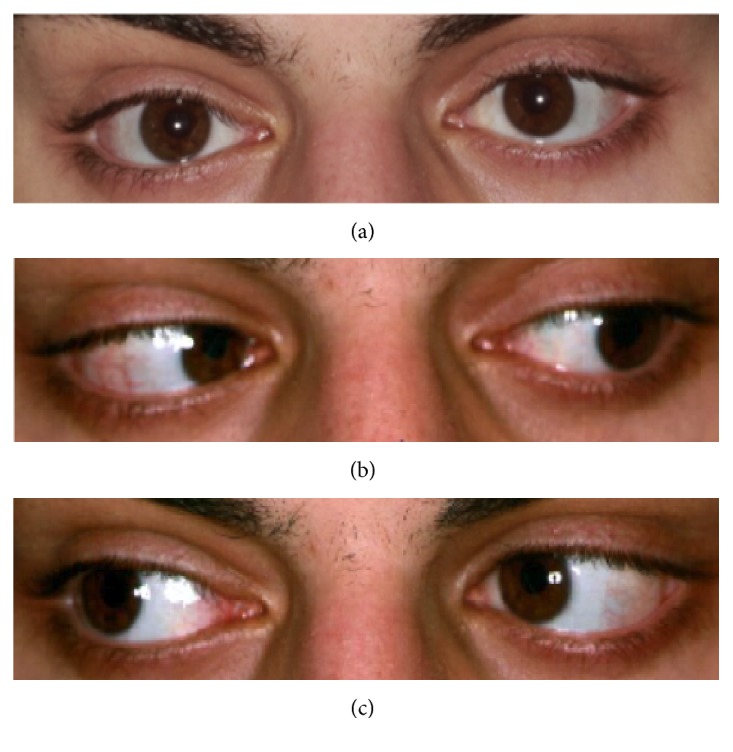
Patient 3, 12 months after Y-split recession. At near fixation (a), left gaze (b), and right gaze (c).

Patient 3 underwent a Y-splitting procedure at the age of 18 years. He had first presented at the age of 18 months with congenital esotropia of about 18 degrees and an amblyopia of the left eye. After sciascopy with atropine, full cycloplegic correction was given, with insignificant reduction of the squint angle. Amblyopia treatment was initiated. Due to poor compliance, the treatment was unsuccessful and subsequently discontinued, as the child developed excentric fixation. Regular examinations followed and at the age of 18 years, the patient inquired about surgical correction of the squint angle, with surgery to be performed only on his left amblyopic eye. Visual acuity for the right eye was 1.25 at distance and at near, for the left eye 0.2 at distance and 0.063 at near. The angle was 42–45 PD at near and 20–25 PD at distance (Table 2). A unilateral Y-splitting procedure of the left medial rectus was performed. At the last FU at 12 months, distance/near disparity was resolved. An esotropia of the left eye with an angle of 18 PD at near and 16–18 PD at distance remained, but the patient was satisfied with the cosmetic result (Figure 2). Binocular functions could not be demonstrated. Motility was good in both eyes.

### 3.4. Patient 4

Patient 4 underwent a bilateral Y-splitting procedure at the age of 10 years.

She suffered from an infantile alternating esotropia with nystagmus, and V-symptom and strabismus sursoadductorius were seen on both eyes. At the age of 4, this had been treated surgically with a 5 mm medial rectus recession, and a 7 mm lateral rectus resection in the right eye and a bilateral inferior oblique recession. 5 years later, wearing full cycloplegic refraction, she presented with a persisting squint angle of 45 PD at near and 30 PD at distance as well as a sursoadduction of the left eye. Snellen acuity was 1.0 at distance and at near OU. She underwent bilateral Y-split recession of the medial rectus muscles in combination with a revision of the inferior oblique muscle of the left eye. 1 week after surgery, the angle decreased to 38 PD at near and 25 PD at distance (Table 2). At the last visit 84 months after surgery, no distance/near disparity was seen. An esotropic angle of 12° at distance and at near remained. A vertical deviation due to the DVD of up to 5° was present on right fixation. The patient was satisfied with the current result, but there is room for improvement: The patient was advised that the remaining esotropia might be addressed by resection surgery on the previously untouched left lateral rectus muscle. However, the patient preferred to force left fixation by blurring the right eye for distance (+3 dpt). Bagolini test remained negative. Motility was good at the last available FU at 3 months.

## 4. Discussion

Splitting procedures have a long history in strabismus surgery. Splitting of the lateral rectus muscle in Duane syndrome has been suggested by Jampolsky as early as 1980 [9]. Here, a leash effect is created to prevent up- or downshoot phenomena in side gaze. In this indication, Y-splitting has recently resurfaced as a valid surgical option [10–13]. Y-splitting procedures have also been suggested in the treatment of oculomotor palsy or cranial dysinnervation syndromes [10, 14].

In the 1990s, splitting of the medial rectus muscle was introduced as a treatment option in patients with a larger strabismus angle at near than at distance (convergence excess) [1]. Bagolini et al. suggested that in patients with varying angles of esotropia, splitting of the medial rectus is an alternative treatment option to the posterior Faden fixation surgery propagated by Cüppers [2]. Since then, other authors have supported the applicability of the Y-splitting procedure in the treatment of esotropia with a larger angle at near than at distance [3–6]. While both surgical procedures reduce the lever arm of the muscle and thus the torque, in the “Fadenoperation,” the medial rectus muscle is fixed to the globe by a fixation suture placed behind the equator of the eye. In contrast, the insertion of the medial rectus muscle remains anterior of the equator in the medial rectus Y-splitting procedure [3–5]. Both methods can be combined with a recession of the medial rectus muscle.

Compared to posterior fixation surgery, Y-splitting surgery results in a larger reduction of the maximal angle at near and in a narrower distribution of the final angle at distance and at near around “0” [7]. Hoerantner et al. have reported the Y-splitting procedure to result in fewer side effects [4, 7]. While the posterior fixation technique harbors a higher risk of perforation [4, 15] and thus retinal detachment, this has not been reported for the Y-splitting procedure.

Another recently propagated surgical techniques to correct distance/near disparity are recessions of the medial rectus muscles where the new insertion is created in a slanting manner [16–18]. These techniques are generally applied bilaterally and reduce disparity by a similar amount [7, 19].

Our small retrospective case series shows that a Y-splitting procedure of the medial rectus resolves the disparity between large angles at near and small angles at distance even when it is done as a secondary and/or as a unilateral procedure. To our knowledge, this has not been reported before.

The reduction of the distance/near disparity may take longer than 3 months but was seen in all patients at final FU >1 yr. This is in agreement with reports of medial rectus-augmented recessions, slanted recessions, or recessions with posterior fixation sutures, where long-term results show an increased effect even after one year [19].

Thus, all our patients had a reduction of their esotropia with a larger effect on the angle at near (average reduction: 38.25 PD) than at distance (average reduction: 20.75 PD, Table 2). Of the four patients, two had a residual angle of 10 PD or less: patient 2 was orthotropic at the final FU and patient 1 had an esotropia of 10 PD on the left fixation which was assured by blurring of the right eye with plus lenses. The remaining 2 patients had a residual esotropia of 16–20 PD and were thus undercorrected in regard to their esotropia. This could have been addressed with an additional resection of the lateral rectus muscle, but both patients elected no further surgery, as they were satisfied with the result. As both patients suffered from congenital esotropia and patient 3 had an additional deep amblyopia, a better sensorineural outcome was not expected with further surgery.

In Y-split recession of the medial rectus muscle as a primary surgery, a concomitant resection of the lateral rectus has been shown to result in larger effects than Y-splitting alone [4]. Our case series suggests that in large angles and unilateral surgery, it is effective to combine a Y-split recession of the medial rectus muscle with a resection of the lateral rectus muscle.

An advantage of the Y-split recession is the lack of a posterior fixation between the extraocular muscle and the sclera and thus no restriction of eye movement. Another advantage is that in a Y-splitting procedure, the medial rectus is reattached anterior to the equator, facilitating revision surgery, if necessary. The mechanism of the Y-splitting procedure can be seen in Figure 1 where the new attachment sites reduce the lever arm of the rectus muscle and thereby the torque. It is not due to a posterior fixation (e.g., due to secondary scarring) as the lack of posterior scarring has been confirmed in patients who have needed a revision of their Y-splitting procedure [7, 20].

To our knowledge, this is the first report on Y-split recessions as a second and/or unilateral intervention (last PubMed search: 22.02.2017).

## 5. Conclusions

Y-splitting procedures offer an alternative method to address esotropia with larger angles at near than at distance. It reliably reduces distance/near disparity also in cases of reoperation and in unilateral surgery. Its effects may take a little longer to stabilize than with traditional recession or resection surgery, but its long term effects are stable. Residual esotropia may be addressed by lateral rectus resection.

## Figures and Tables

**Table 1:** Patient characteristics and surgical data.

Patient	1		2		3		4	
Age at surgery	23		15		18		10	
Previous strabismus surgery	Yes		Yes		No		Yes	
Side of surgery	Right		Left		Left		Bilateral	
Axial length (mm) OD/OS	23.3	22.69	22.63	22.59	24.47	23.35	22.82	23.09
rA	10.8		10.5		10.6		OD 7.75	OS 9.25
rB	8.24		8		9.2		OD 8.2	OS 9.5
Concomitant lateral rectus resection	5 mm		5 mm		No		No	

**Table 2:** Strabismus angles in prism diopters.

Patient			1		2		3		4		Av.
FU (months)			36		60		12		60		42
			Angle at near	Angle at distance	Angle at near	Angle at distance	Angle at near	Angle at distance	Angle at near	Angle at distance	Disparity
Angle	Baseline		66	55	51	27	45	25	45	30	17
	FU	1 week	30	16	16	−3	35	18	38	25	—
		3 months	35	35	20 (@ 6mo)	5 (@ 6mo)	20	10	25	16	—
		Last FU	16	16	0	0	18	18	20	20	0
∑ reduction of disparity			11		24		20		15		—

Av: average.
